# Exploring spatial dynamics of trauma and substance use among suicide deaths in the United States (2017–2021)

**DOI:** 10.1186/s40621-025-00574-0

**Published:** 2025-05-01

**Authors:** Bianca D. Smith, Kechna Cadet, Terrinieka W. Powell

**Affiliations:** 1 Department of Mental Health, Johns Hopkins Bloomberg School of Public Health, Baltimore, MD USA; 2 Department of Epidemiology, Columbia University Mailman School of Public Health, New York, NY USA; 3 Department of Population, Family and Reproductive Health, Johns Hopkins Bloomberg School of Public Health, Baltimore, MD USA

**Keywords:** Suicide, Trauma, Substance use, Spatial epidemiology, Hot spot

## Abstract

**Background:**

Suicide remains a significant cause of death in the United States. Traumatic events, such as experiences of violence, financial loss, and mental illness, significantly increase an individual’s risk of suicide. Substance use, often used as a coping mechanism for trauma, frequently occurs alongside these events. Geographic patterns of trauma and substance use may reveal underlying factors that contribute to suicide rates across the nation.

**Methods:**

Data from the National Violent Death Reporting System (NVDRS), collected between 2017 and 2021, was used to examine spatial relationships between traumatic events and substance use among suicides. Spatial autocorrelation was used to assess global spatial dependence of traumatic events among suicide deaths. Additionally, hot spot analyses were conducted to pinpoint regions with significantly elevated or reduced experiences of trauma. Colocation analyses were conducted to identify areas where traumatic events and substance use co-occur spatially.

**Results:**

Traumatic events among suicides exhibited geographic clustering. Spatial clusters of traumatic events were identified in specific regions across the U.S. and its territories. Hot spots were predominantly observed in Western and Midwestern areas, while more cold spots were found in Southern regions. Additionally, colocation analysis revealed that Midwestern counties had a higher likelihood of experiencing traumatic events in conjunction with substance use history among suicide decedents.

**Conclusion:**

Clustering patterns may provide insight on underlying mechanisms that have significant impacts on suicide outcomes. The colocation analysis helps reveal patterns of spatial clustering, shedding light on potential risk factors or shared characteristics in those areas. By examining both global and local spatial patterns, researchers gain insights into the distribution of trauma and substance use-related incidents and their association with suicide.

## Introduction

Over the past five years, suicide has consistently been a leading cause of death in the United States (U.S.) [1]. After a two-year decline from 2018 to 2020, suicide rates rose again following the COVID- 19 pandemic [1]. Experiences of trauma (i.e., traumatic events) and substance use disorders are complex mental health-related issues that can influence suicidality among adult populations [2]. Individuals who have experienced traumatic events, such as family problems, academic failures, or financial strains, are at a significantly higher risk of developing suicidal ideation and engaging in suicidal actions [3, 4]. Research indicates that the cumulative effect of multiple traumatic experiences can also increase the risk of developing a substance use disorder [5]. Substance use disorders (SUD) are often preceptors for suicide outcomes (e.g., suicidal thoughts, behaviors, and suicide attempts) [6, 7]. For example, individuals with substance use disorders (SUDs) are at least twice as likely to report having attempted suicide at some point in their lives compared to those without SUDs [8, 9]. When trauma and substance use co-occur, they exacerbate an individual’s vulnerability to suicide. For example, trauma can exacerbate substance use, and vice versa, leading to heightened emotional distress and hopelessness [10, 11]. The combined burden of trauma and substance use can overwhelm coping mechanisms, increasing suicidal thoughts and behaviors [9].

Geography is a significant factor in population-level trends in suicide mortality [4]. Recent studies have shown that various environmental characteristics, such as changes in demographic trends and the built environment, can influence the spatial clustering of suicide [12, 13]. Furthermore, the COVID- 19 pandemic introduced new spatial dynamics, with some areas experiencing fluctuations in suicide rates due to economic and social stressors [12]. Suicide rates have been notably higher in the Southern, Midwestern, and Western states in the U.S. Moreover, geographic disparities in suicide rates may be reflective of spatial distributions of other health outcomes [4]. Interestingly, suicide mortality spatial trends mirror patterns seen in substance use disorders (SUDs), with rural areas disproportionately affected [12].

The co-occurrence of traumatic events and substance use may play a role in shaping suicide patterns. While both trauma and substance use are recognized risk factors, less is known about the spatial patterns of traumatic events and whether the co-occurrence of traumatic events and substance use spatially impacts the distribution of suicide [14, 15]. Understanding these spatial patterns is crucial for developing targeted interventions and policies aimed at reducing suicide mortality, particularly in high-risk areas. This is particularly important in creating a more nuanced and comprehensive understanding of suicidality and moves the field beyond a ‘one size fits all’ intervention approach in tackling suicide as a public health issue.

The purpose of this study was to examine the spatial pattern of traumatic events among suicides and examine co-occurring patterns of trauma and substance use. Geospatial data allows researchers to identify hotspots where trauma, substance use, and suicidal behaviors are more prevalent. This spatial analysis will provide insights on the geographical variations in suicide risk factors. Understanding these spatial dynamics is essential for creating timely and effective public health responses. This research contributes to the growing body of knowledge on the spatial patterns of suicide, trauma, and substance use. By leveraging geospatial data and advanced analytical techniques, we aimed to identify high-risk areas and inform the development of targeted, evidence-based interventions to reduce suicide mortality and improve mental health outcomes across diverse populations.

## Methods

## Data source

The National Violent Death Reporting System (NVDRS) is an active national surveillance system that collects data on violent death (e.g., suicide, homicide) across 50 U.S. states, Washington D.C., and Puerto Rico [16]. NVDRS data are collected by medical examiners, coroners, and law enforcement personnel using medical examiner and toxicology reports, death certificates, and police reports [16]. Data is abstracted by the primary source data provider or transferred from the data provider office. Data is entered manually or electronically imported [16]. Access to restricted data was approved by the CDC’s NVDRS Restricted Access Data Review Committee. The NVDRS uses a case definition of suicide developed by the CDC to account for differences in classification from coroners and medical examiners [16]. Suicide is defined as “a death resulting from the intentional use of force against oneself,” and includes various scenarios under which a suicide could occur (e.g., assisted suicide, engaging in suicidal behavior that results in death, intentional injury that results in death, etc.) [16]. Deaths were classified as suicides when there was substantial evidence indicating that the use of force was intentionally self-inflicted [16]. Comprehensive details on the procedures for coding suicide deaths can be found elsewhere [17].

## Study population

Our sample was restricted to suicide deaths among those who were 18 years and older from 2017 to 2021 (*N* = 171,615) across the participating states and territories. NVDRS data collection has shown significant geographic variations over the years, with 37 states reporting in 2017, 41 states in 2018, 44 states in 2019, and all 50 states reporting in both 2020 and 2021 [16]. Table 1 provides information on the distribution of demographics, suicide-related behaviors, traumatic events, and history of substance use over time. Across the five years, trends show that most suicide decedents were on average 48 years old at the time of death, non-Hispanic White, and biologically identified as male. Suicide characteristics showed that, on average, roughly 30% of the decedents had a history of suicidal thoughts, and approximately 17% had a previous suicide attempt before their death. More than 40% of the sample was noted as having a mental health problem (e.g., major depression, neurodevelopmental disorders, dementia, etc.) across all years. On average, the number of traumatic events was less than 2, with intimate partner problems and having a physical health problem being the most common events reported.

**Table 1:** Characteristics of suicide deaths from 2017–2021 across the united States (*N* = 171,615)

Variables	2017 (n = 28,644)	2018 (n = 33,677)	2019 (n = 32,190)	2020 (n = 37,302)	2021 (n = 39,802)	Total (n = 171,615)
**Age**						
Mean (SD)	47.6 (18.1)	47.8 (18.1)	48.0 (18.2)	47.5 (18.7)	47.3 (18.7)	47.6 (18.4)*
**Race**						
White	25,087 (87.6%)	29,391 (87.3%)	27,911 (86.7%)	32,037 (85.9%)	33,739 (84.8%)	148,165 (86.3%)*
Black/African American	1,829 (6.4%)	2,242 (6.7%)	2,122 (6.6%)	2,748 (7.4%)	3,203 (8.0%)	12,144 (7.1%)
American Indian/Alaska native	365 (1.3%)	410 (1.2%)	408 (1.3%)	485 (1.3%)	593 (1.5%)	2,261 (1.3%)
Asian/Pacific Islander	778 (2.7%)	937 (2.8%)	963 (3.0%)	1,085 (2.9%)	1,151 (2.9%)	4,914 (2.9%)
Other/unspecified	284 (1.0%)	321 (1.0%)	336 (1.0%)	332 (0.9%)	425 (1.1%)	1,698 (1.0%)
Two or more races	300 (1.0%)	340 (1.0%)	392 (1.2%)	432 (1.2%)	477 (1.2%)	1,941 (1.1%)
Unknown	1 (0%)	36 (0.1%)	58 (0.2%)	183 (0.5%)	214 (0.5%)	492 (0.3%)
**Ethnicity**						
Not Hispanic or Latino	26,347 (92.0%)	31,002 (92.1%)	29,708 (92.3%)	34,040 (91.3%)	36,133 (90.8%)	157.230 (91.6%)*
Hispanic or Latino	2,119 (7.4%)	2,553 (7.6%)	2,429 (7.5%)	3,169 (8.5%)	3,604 (9.1%)	13,874 (8.1%)
Unknown	176 (0.6%)	116 (0.3%)	52 (0.2%)	89 (0.2%)	62 (0.2%)	495 (0.3%)
**Biological sex**						
Male	22,310 (77.9%)	26,400 (78.4%)	25,368 (78.8%)	29,867 (80.1%)	31,958 (80.3%)	135,903 (79.2%)*
Female	6,333 (22.1%)	7,275 (21.6%)	6,822 (21.2%)	7,433 (19.9%)	7,839 (19.7%)	35,702 (20.8%)
Unknown	1 (0.0%)	2 (0.0%)	0 (0.0%)	2 (0.0%)	4 (0.0%)	9 (0.0%)
**Relationship status at the time of incident**						
Currently in a relationship	11,724 (41.2%)	13,889 (42.2%)	13,425 (41.9%)	14,456 (41.0%)	15,432 (40.6%)	68,926 (41.4%)*
Not currently in a relationship	2,525 (8.9%)	2,523 (7.7%)	2,687 (8.4%)	3,246 (9.2%)	3,879 (10.2%)	14,860 (8.9%)
Unknown	14,174 (49.9%)	16,472 (50.1%)	15,910 (49.7%)	17,573 (49.8%)	18,738 (49.2%)	82,867 (49.7%)
**Suicide characteristics** ^**a**^						
Previous suicide attempt	5,258 (18.4%)	6,076 (18.0%)	5,730 (17.8%)	5,757 (15.4%)	5,933 (14.9%)	28,754 (16.8%)*
History of suicidal thoughts	8,672 (30.3%)	10,400 (30.9%)	10,545 (32.8%)	11,071 (29.7%)	11,459 (28.8%)	52,147 (30.4%)*
**Substance use characteristics** ^**a**^						
Alcohol dependence or alcohol problem	4,950 (17.3%)	5,793 (17.2%)	5,746 (17.9%)	5,980 (16.0%)	6,183 (15.5%)	28,652 (16.7%)*
Non-alcohol related substance abuse	4,721 (16.5%)	5,248 (15.6%)	5,175 (16.1%)	5,711 (15.3%)	6,201 (15.6%)	27,056 (15.8%)*
**Traumatic events** ^**a**^						
Total number of traumatic events Mean (SD)	1.58 (1.30)	1.53 (1.30)	1.53 (1.28)	1.44 (1.32)	1.39 (1.28)	1.49 (1.30)*
Currently having a mental health problem	13,015 (45.4%)	14,808 (44.0%)	14,080 (43.7%)	15,113 (40.5%)	16,509 (41.5%)	73,525 (42.8%)*
Intimate partner problem	7,086 (24.7%)	8,132 (24.1%)	7,731 (24.0%)	8,458 (22.7%)	8,531 (21.4%)	39,938 (23.3%)*
Family relationship problem	2,294 (8.0%)	2,495 (7.4%)	2,153 (6.7%)	2,258 (6.1%)	2,319 (5.8%)	11,519 (6.7%)*
Other relationship problem	538 (1.9%)	559 (1.7%)	554 (1.7%)	681 (1.8%)	689 (1.7%)	3,021 (1.8%)
History of abuse or neglect as a child	259 (0.9%)	301 (0.9%)	322 (1.0%)	346 (0.9%)	340 (0.9%)	1,568 (0.9%)
Physical fight between two people	242 (0.8%)	291 (0.9%)	274 (0.9%)	324 (0.9%)	323 (0.8%)	1,454 (0.8%)
Disaster exposure	43 (0.2%)	66 (0.2%)	38 (0.1%)	1,485 (4.0%)	979 (2.5%)	2,611 (1.5%)*
Homeless	403 (1.4%)	468 (1.5%)	485 (1.6%)	579 (1.7%)	588 (1.6%)	2,523 (4.1%)*
Housing instability	0 (0.0%)	1 (4.8%)	722 (4.8%)	1,116 (4.1%)	1,093 (3.6%)	2,932 (4.1%)*
Contributing criminal legal problem	2,121 (7.4%)	2,358 (7.0%)	2,267 (7.0%)	2,175 (5.8%)	2,331 (5.9%)	11,252 (6.6%)*
Civil legal problems	836 (2.9%)	1,113 (3.3%)	1,065 (3.3%)	921 (2.5%)	854 (2.1%)	4,789 (2.8%)*
Contributing physical health problem	5,827 (20.3%)	6,531 (19.4%)	6,257 (19.4%)	6,417 (17.2%)	6,898 (17.3%)	31,930 (18.6%)*
Job problem	2,577 (9.0%)	2,864 (8.5%)	2,655 (8.2%)	2,887 (7.7%)	2,725 (6.8%)	13,708 (8.0%)*
Financial problem	2,322 (8.1%)	2,698 (8.0%)	2,377 (7.4%)	2,111 (5.7%)	1,854 (4.7%)	11,362 (6.6%)*
School problem	171 (0.6%)	200 (0.6%)	181 (0.6%)	133 (0.4%)	167 (0.4%)	852 (0.5%)*
Eviction or loss of housing	1,021 (3.6%)	1,123 (3.3%)	1,018 (3.2%)	873 (2.3%)	839 (2.1%)	4,874 (2.8%)*
Suicide of friend or family contributed to death	623 (2.2%)	774 (2.3%)	694 (2.2%)	688 (1.8%)	705 (1.8%)	3,484 (2.0%)*
Other death of friend or family	1,785 (6.2%)	2,038 (6.1%)	1,775 (5.5%)	1,856 (5.0%)	2,199 (5.5%)	9,653 (5.6%)*

^a^ Reflects the number and percentage of participants answering “yes” to this question

*p < 0.05. Measuring whether there were significant changes in frequency over time

## Measures

### Demographics

Data on the decedent’s demographic characteristics included the year of incident/death, age, race, biological sex, ethnicity, and relationship status. Demographic information was abstracted from death certificates, law enforcement and coroner/medical examiner reports.

### Suicide characteristics

Suicide characteristics were captured using two items. *History of suicidal thoughts* were reported when the narrative reports indicated the “victim had a history of suicidal thoughts or plans (either through verbal, written, or electronic disclosure of plan or thoughts).” *Previous suicide attempts* were reported when the “victim has a history of attempting suicide before the fatal incident.”

### Traumatic events

Traumatic events are broadly defined as events that can cause significant distress or physical and emotional harm [18]. The measurement of traumatic events in this study was guided by the Trauma History Screen (THS). The THS is a self-report measure of trauma experiences related to 13 events (e.g., physical assault, sudden loss of home, death of a family member or friend) that can be used across a wide population [19]. We expanded the number of traumatic events to capture experiences in different domains (e.g., household, school, relationships). Eighteen items were used to capture traumatic events in this sample. These traumatic events were reported as events that appear to have contributed to the suicide. The included events were: *intimate partner problem*,* family relationship problem*,* other relationship problems*,* history of abuse or neglect as a child*,* physical fight between two people*,* disaster exposure*,* homelessness*,* housing instability*,* contributing criminal legal problems*,* civil legal problems*,* contributing physical health problem*,* job problem*,* financial problem*,* school problem*,* eviction or loss of housing*,* suicide of friend or family contributed to death*,* other death of friend or family*,* and current diagnosed mental health problem*. NVDRS abstractors indicated whether the listed events were reported in the decedents’ narrative reports using a binary coding system (*1: Yes and 0: No*,* not available*,* unknown*). Traumatic events were quantified as a continuous variable by summing the responses to each indicator, with a possible maximum score of 18.0. For the colocation analysis, this variable was measured categorically (*1: traumatic event total was ≥ 1.0 and 0: traumatic event total = 0.0*).

### Substance use

Substance use was measured using two items. *Alcohol use* was measured using the item that captured whether the decedent had a “alcohol dependence or alcohol problem.” *Other substance use* (e.g., illicit drugs, prescription medications, inhalants) was measured using the item that captured “non-alcohol related substance abuse problem.” These were coded as binary variables (*1: Yes and 0: No*,* not available*,* unknown*). The final substance use variable was a combination of the two items, indicating whether the decedent had a history of alcohol or substance use (*1: Yes and 0: No*,* not available*,* unknown*).

## Statistical analysis

First, we examined the frequency and trends of demographic and suicide characteristics, traumatic events, and history of substance use across the 5-year timeframe. Chi-square and t-tests were conducted to measure significant changes in frequencies across time for demographic and behavioral variables. We restricted our data to suicides occurring between 2017 and 2021 to account for changes across geographic boundaries. NVDRS data was geocoded to the county level using the boundaries from the 2017–2021 American Community Survey. Approximately 0.75% (*n* = 1,292) of the data could not be matched geographically, so the final sample for geospatial analyses was 171,323 suicide deaths. Spatial autocorrelation was conducted to examine the spatial pattern of traumatic events among suicide decedents to capture whether counties with a similar number of traumatic events among suicide decedents tend to be clustered together. Using the k-nearest neighbor approach, the global Moran’s Index was calculated for the number of traumatic events across the participating states for each year. We also used hot spot analysis to identify local clusters of traumatic events. This analysis produced a Getis-Ord Gi* statistic, a measure of local spatial autocorrelation, to identify significantly higher or lower values of traumatic events across space [20–22]. Each suicide death had a calculated z-score and p-value that indicated whether the location of the death is a hot or cold spot for trauma. Next, we examined the global and local spatial associations between traumatic events and history of substance use using colocation analysis. Colocation analysis measures the patterns of association between categories (i.e. the likelihood of the two categories occurring in the same locations) [23, 24]. In our study, we were interested in the geographical relationship between trauma and substance use, focusing on whether trauma is spatially influenced by substance use as it relates to suicide deaths. Colocation analysis is a growing geospatial tool within public health research and has often been used to understand patterns of crime and injury-related outcomes [25–27]. Colocation analysis uses point data; therefore county polygons were converted to XY-point data using the latitude and longitude coordinates. Colocation analysis settings were time-bound with a 1-year interval, specifically defined as a ‘before’ temporal relationship. This approach allows us to use a space-time window to understand colocation patterns. In practical terms, each data point only considers events that occurred within a 1-year window before the date of death and are geographically proximate. The date of death was reported by year only. Given the variations in state reporting over the study period, using a 1-year window may minimize reporting bias by only considering the previous year’s reported data. Additionally, we employed a distance band neighborhood type, analyzing each county within the context of neighboring counties located within a calculated sphere of influence. Analyses were conducted in STATA 8.0 and ArcGIS Pro 3.3.

## Results

### Spatial autocorrelation: clustering of traumatic events

Moran’s I, a measure of global spatial dependence, measured the number of traumatic events across the study area. Table 2 shows the results from the spatial autocorrelation. Results show that there is a global association between traumatic events and geographic space among suicides. Traumatic events exhibit non-random patterns, showing a clustered pattern across all years. Said another way, traumatic events are occurring in specific areas across the nation.

**Table 2:** Spatial autocorrelation of traumatic events

	2017 (n= 28,517)	2018 (n= 33,498)	2019 *(*n= 32,032)	2020 (n= 36,916)	2021 (n= 39,360)
Moran’s I	0.162088	0.180484	0.156946	0.199496	0.195350
P-value	< 0.001	< 0.001	< 0.001	< 0.001	< 0.001
Dispersion type	Clustered	Clustered	Clustered	Clustered	Clustered

### Hot/Cold spots

**Fig. 1:**
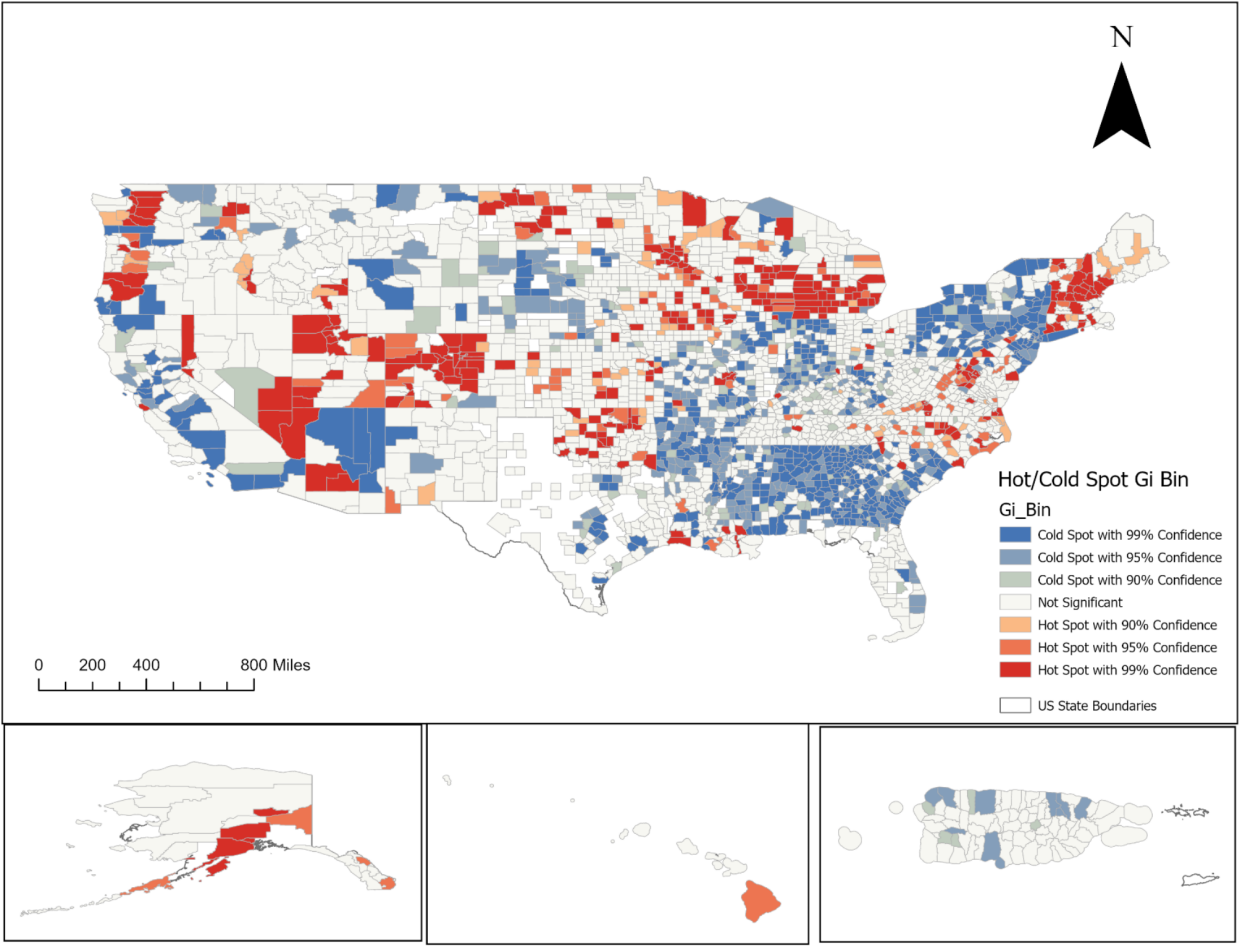
Hot and cold spot of traumatic events among suicide decedents, 2017–2021

Figure 1 displays the distribution of hot and cold spots of traumatic events. We primarily observed significant hotspots (areas with significantly higher amounts of traumatic events among suicide decedents) in Midwestern areas, such as Illinois, Michigan, and Minnesota. In the Western areas, we observed county level hot spots in Alaska, Washington, and Arizona. Few areas in the Eastern region (i.e., counties within New Hampshire, Maine, and Vermont) contained significant hot spots. Cold spots, areas with significantly lower levels, were mainly located in the southern region of the U.S. For example, we observed that most of the counties in Georgia and Alabama were considered cold spots. Areas in the northeast were also displaying patterns of cold spots, with a large portion of New York counties having significantly lower amounts of traumatic events among suicide decedents.

### Colocation of trauma and substance use

Table 3 presents the global colocation quotients (GCLQ) between the categories of trauma and substance use. Our global colocation analysis revealed significant spatial relationships between trauma and substance use. The global colocation quotient was significant (1.037, *p* = 0.02) between the presence of trauma and history of substance use, showing that counties reporting traumatic events among suicide decedents tended to be near counties where decedents also had a history of substance use. In other words, having a history of substance use is more likely to occur near areas with experiences of trauma among suicides. Conversely, counties where decedents reported no history of substance use tended to be isolated from areas where trauma was present (GCLQ = 0.985, *p* = 0.02). These varied spatial relationships suggest that the distribution of trauma and substance use is not random but follows distinct patterns. These patterns can be effectively visualized using local colocation analysis.

**Table 3:** Global colocation between trauma and substance use among suicides

Category A	Category B	Global colocation quotient	Type of relationship	P-value
Trauma present	History of substance use	1.04	Colocated	0.02
Trauma present	No history of substance use	0.99	Isolation	0.02
Absence of trauma	History of substance use	0.87	Isolation	0.02
Absence of trauma	No history of substance use	1.08	Colocated	0.02

**Fig. 2:**
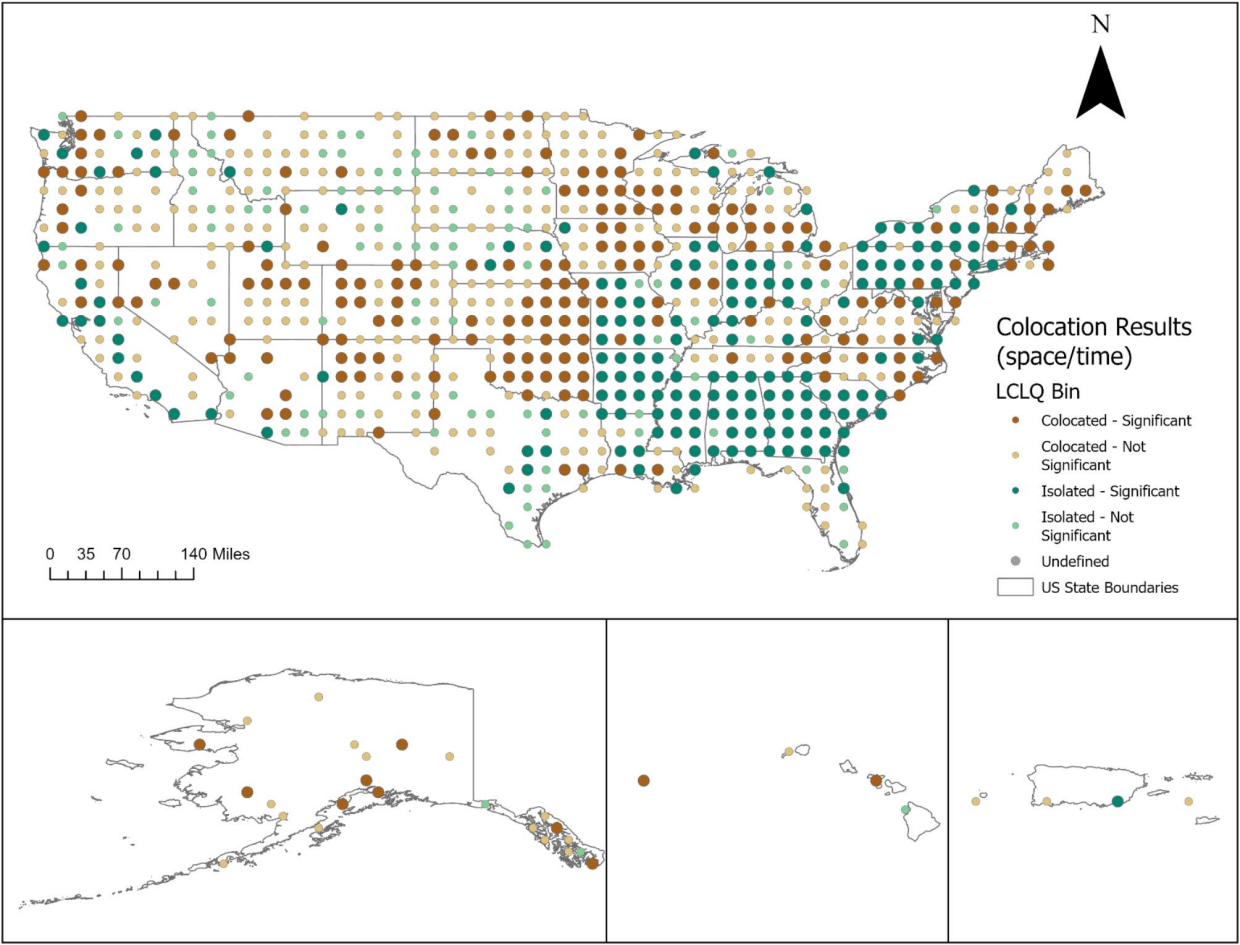
Local colocation between traumatic events and substance use among suicide decedents, 2017–2021

Figure 2 illustrates the distribution of local colocation patterns across the study area. Notably, significant colocation between trauma and substance use emerges prominently in the Midwestern and Western regions of the U.S. In the Midwest, we observe a concentrated area among counties with suicide decedents who experienced traumatic events and had a history of substance use. In the Western regions, the colocation patterns are more scattered, indicating a broader distribution of trauma and substance use across multiple counties. This suggests that while there are significant colocated areas, the relationship between trauma and substance use is not confined to a single area but rather spread out across the region. Conversely, isolation patterns predominantly manifest in the southern regions and parts of the Northeast. In Southern states, such as Alabama and Georgia, we observe only isolated areas. Similarly, Northeastern states, New York and Pennsylvania, exhibit more isolation patterns compared to the New England states.

Additionally, certain areas within the Midwest exhibit isolation, where suicide decedents with trauma are notably distant from regions where decedents reported substance use histories.

## Discussion

This study sought to understand the geographic distribution of trauma among suicide deaths, and the spatial relationship between common suicide risk factors. Findings showed that experiences of trauma tend to occur in distinct clusters across geographic space. Hotspots were primarily observed in Midwestern areas (e.g., Illinois, Michigan, Minnesota) and Western areas (e.g., Alaska, Washington, Arizona). Cold spots were mainly located in the southern U.S. (e.g., Georgia, Alabama) and parts of the Northeast (e.g., New York). Lastly, there was a global and local spatial relationship between trauma and substance use among suicide deaths.

We found that, on average, less than two traumatic events were reported as possible contributors to suicides. This finding indicates that multiple experiences of trauma are relatively uncommon among individuals who die by suicide. Consistent with other research, having a mental health issue and intimate partner problems were common events among suicide decedents [3, 8, 17, 28]. However, physical health problems, which are rarely assessed during trauma screenings, were also highly reported by suicide decedents. Having mental or physical health problem and experiencing intimate partner problems may be enough of a traumatic event to influence a person’s suicidal behavior [29]. The COVID- 19 pandemic occurred during our study period; large-scale events, such as a pandemic, can impact the geographic distribution of traumatic events [30]. Structural policies and regulations, such as quarantines and “stay-at-home orders”, increased exposure to intimate partner violence [31]. Similarly, mental health issues worsened across the nation, which led to significant increases in suicides and substance use disorders [32, 33].

However, when trauma occurs, it happens in specific geographic areas. Our findings showed a spatially dependent pattern of trauma among suicide deaths, highlighting that the geography of trauma is a crucial factor in better understanding suicidality. Further investigation revealed that individuals who died by suicide experienced higher amounts of traumatic events in the Midwest and West. Our findings support previous research indicating that rurality plays a significant role in shaping state-level variations in suicide cluster patterns [4, 34, 35]. However, there were counties in urban areas in the Midwest with significantly higher levels of trauma, indicating that trauma among suicides has specific geographic differences. We also observed a pattern of cold spots in the South, where trauma was not significantly clustered among suicide decedents in this region. Suicides were occurring, however, the number of traumatic events may not significantly impact the risk of suicide in this region. Future research could benefit from examining how spatial relationships vary according to the type of traumatic event. Understanding the spatial differences in the types of trauma may help explain the evolving regional variations in suicide trends.

The co-occurrence of trauma and substance use can magnify the risk of suicide among adults [2]. In this study, the co-occurring spatial relationship between trauma and substance use varied based on the presence or absence of substance use. Our findings suggest that when trauma is present among suicide decedents, it tends to be near areas where decedents also have a history of substance use. We also observed local variations across the nation. These variations highlight the importance of considering local differences in both trauma and substance use when addressing suicide prevention. Substance use spatial trends show a distribution similar to suicide, often occurring in the West and more rural areas [36]. Notably, in the Midwest, there are more instances of colocated trauma and substance use among suicide deaths. This observation may explain why the Midwest does not appear as a hotspot for suicide clusters. While existing research often focuses on demographics and suicide-specific characteristics among decedents to understand spatial suicide trends, our study expanded to explore the role of other common risk factors, such as trauma and substance use. Our findings suggest that while rurality remains a prominent geographic factor in most areas, urbanicity may also play a role in shaping suicide trends. Future research should continue to investigate these spatial dynamics to gain insights that can guide early detection and suicide prevention efforts. For example, identifying areas with high co-occurrence of trauma and substance use can prompt targeted screening for suicide. Furthermore, understanding where this colocation is occurring can strategically aid in allocating mental health resources based on spatial disparities.

## Limitations

There are several limitations to this study. First, we used counties as the geographic unit of analysis. While counties are typical in spatial studies, smaller units (e.g., block groups) provide a more precise understanding of these relationships. Although our findings are significant, examining smaller geographic areas would allow researchers and policymakers to pinpoint neighborhoods or districts experiencing higher levels of trauma. However, we employed multiple geospatial methods to capture spatial dependence using both global and local measures, which can inform future research and policy recommendations by identifying high-risk areas. Next, our study relied on data abstracted from police reports and death certificates. These sources may be less accurate or more incomplete regarding the circumstances before death, which are crucial for effective intervention. Information about the history of trauma and substance use is often collected retrospectively from others (e.g., family members, neighbors) rather than the individual, making it subjective and potentially incomplete. Additionally, suicides are often misclassified on death certificates, leading to potential underreporting of deaths in our sample and impacting the geographical distribution [37]. Similarly, there were changes in the number of states reporting data over the study period. Although we incorporated a space-time window to account for these changes, our findings may be biased in one direction where the increase in data reporting could influence the observed trends. Likewise, although we used counts that were spatially weighted, we did not use population-adjusted rates, which may be more representative of the true distribution of the data. This approach could provide a more accurate reflection of the underlying population dynamics and trends.

However, a strength of this research is that we expanded the number of traumatic events to capture a more holistic view of experiences prior to suicide. Lastly, we did not examine macro-level determinants that influence suicidality. Economic disparities can exacerbate stress and limit access to mental health resources, while policies related to housing, healthcare, and social services can either mitigate or amplify these effects [4]. Structural racism, manifesting through discriminatory practices and systemic inequalities, can create environments where certain populations are more vulnerable to trauma and substance use [4]. Segregation spatial patterns may explain why certain regions are more impacted compared to others, highlighting demographic groups that may be disproportionately affected. Understanding these macro-level determinants is crucial for developing comprehensive prevention strategies that address the root causes of suicidality and promote health equity across different communities.

## Conclusion

Using spatial methods to study common risk factors for suicide enhances our understanding of suicidality. By mapping where trauma occurs, we can find areas where interventions might work best, revealing local patterns and risk factors that might otherwise stay hidden. This approach improves our knowledge of how trauma is distributed geographically and helps create targeted strategies to prevent suicide. As a result, public health can use resources more efficiently, design specific interventions, and provide better support for at-risk populations. Ultimately, this spatial analysis leads to more effective public health actions, promoting a proactive approach to suicide prevention and improving mental health outcomes in various communities.

## Data Availability

The dataset analyzed during the current study is available by request via the NVDRS Restricted Access Database application process: https://www.cdc.gov/nvdrs/about/nvdrs-data-access.html.
